# Outcomes of Arteriovenous Fistula Creation in Patients Undergoing Hemodialysis: An Indian Experience

**DOI:** 10.7759/cureus.20921

**Published:** 2022-01-04

**Authors:** Arunesh Gupta, Vineet Kumar, Amit R Peswani, Aneesh Suresh

**Affiliations:** 1 Plastic and Reconstructive Surgery, Topiwala National Medical College and B.Y.L. Nair Charitable Hospital, Mumbai, IND; 2 Plastic and Reconstructive Surgery, Tata Memorial Hospital, Homi Bhabha National Institute, Mumbai, IND

**Keywords:** renal transplantation, chronic kidney disease, predictive factors, haemodialysis, vascular access, arteriovenous fistula

## Abstract

Introduction

Creating an arteriovenous fistula (AVF) to provide a patent and long-term vascular access (VA) for hemodialysis (HD) still remains a challenge. A methodical approach to choosing the appropriate HD access in accordance with patients’ end-stage kidney disease (ESKD) life plan will help them achieve their goals safely. This study summarizes the impact of various factors on the AVF outcomes in an Indian population as well as the necessary considerations before choosing the site of AVF creation.

Materials and methods

This study involved a single-center, retrospective evaluation of all patients who had undergone arteriovenous (AV) access creation for maintenance HD from October 2018 to August 2019 at a center in India.

Results

In our study of 216 cases, the average age at presentation was 43.9 years and the difference in age between the successful and unsuccessful group was not significant. The successful outcomes in males were significantly higher than those in females (p=0.005). The mean venous diameter in the successful group was significantly larger than that in the unsuccessful group. The distal arterial and vein diameter was higher in both males and females of the laborer group compared to the clerical group; however, the outcomes were comparable. The overall complication rate was 22.22%. We had primary patency rates of 83% at the end of one year with a primary failure rate of 8.80%.

Conclusion

Vein diameter was the most important predictive factor for a successful outcome in our study. Factors like age and life expectancy, gender, comorbidities, occupation, and type of anastomosis may not be individually predictive of outcomes but need to be considered before choosing the appropriate site of access creation according to the life plan of the patient. This will reduce morbidity associated with an additional procedure and facilitate the initiation of HD as early as possible. Occupation can be considered as a surrogate for preoperative forearm exercises with the increased caliber of vessels found in people performing heavy/manual labor favoring a more distal AVF creation.

## Introduction

The steady increase in the number of patients requiring maintenance hemodialysis (HD) warrants a logical and systematic approach to create good vascular access (VA) [1]. With an increasing number of patients presenting at a younger age, it is imperative to provide a single VA that is reliable and long-lasting. The VA needs to be tailored to the individual patient, providing continuous viable access for a long duration with minimal complications [2]. Since the appropriate dialysis access aligns with the final modality for kidney replacement therapy (dialysis or transplant), it must be individualized to help each patient achieve their life goals safely [2]. This study summarizes the impact of various factors on the arteriovenous fistula (AVF) outcomes in an Indian population and aspects that need to be considered before choosing the site of AVF creation.

## Materials and methods

Objectives

• Evaluating the impact of age, sex, comorbidities, and occupation on successful AVF maturation.

• Assessing the impact of occupation on vessel caliber and its impact on the outcome.

• Evaluating the effect of the diameter of vessels (arteries and veins) and choosing the type of anastomosis with its impact on the outcome.

This study was a single-center, single-surgeon, retrospective evaluation of patients who had undergone arteriovenous (AV) access creation for maintenance HD in chronic kidney disease (CKD) from October 2018 to August 2019. The data was collected by adhering to the principles outlined in the Declaration of Helsinki 1975 (revised in 2008). 

Inclusion and exclusion criteria

All chronic renal failure (CRF) patients referred from the Department of Nephrology were considered for AVF creation except those who had severe peripheral arterial disease on ultrasound (USG) Doppler. Follow-up details were obtained in collaboration with the HD unit staff as per telephonic calls and outpatient department visits by the patients. The data extracted from hospital records included the patient’s demographic details, comorbidities, site and type of AVF, operative details, patency, and fistula-related complications. 

The parameters assessed during the perioperative period are presented in Table 1.

**Table 1:** Evaluation parameters in the perioperative period GFR: glomerular filtration rate

Preoperative evaluation	Intraoperative evaluation	Postoperative evaluation
Occupation	Diameter of the artery and vein	Any complications in the postoperative period like bleeding, hematoma, infection, thrombosis
Hand dominance	Presence of atherosclerosis	Patency of fistula at 1, 3, 6 months, 1 year, and then yearly thereafter
History of diabetes mellitus, cardiovascular disease, peripheral arterial disease, anticoagulant therapy	Visible arterial pulsations	
Recent puncture near the planned site of fistula creation	Immediate venous dilatation after anastomosis	
Timing of the previous hemodialysis	Presence of thrill after anastomosis	
Physical examination of arterial pulses and venous system		
Allen’s test		
GFR <30 ml/min		
Preoperative documentation of vein and artery diameter by Doppler		

Surgical technique

The following four types of fistulas were performed:

1. Anatomical snuff box

2. Radiocephalic in the wrist and forearm

3. Brachiocephalic

4. Brachiobasilic with one-stage basilic vein transposition

The site of fistula creation was chosen based on the findings on preoperative USG Doppler. In the absence of arterial pathologies like atherosclerosis/narrowing and a vein diameter of more than 2 mm, fistula creation can be attempted as distally as feasible. All surgeries were performed under local anesthesia using 2% lignocaine and 1:2,00,000 adrenaline (containing 21.3 mg/ml lignocaine hydrochloride and 0.009 mg/ml adrenaline bitartrate) or regional block, with loupe magnification using microvascular instruments.

A) Distal Radiocephalic AVF (RCAVF) Creation

Identification of the cephalic vein and superficial branch of the radial nerve is a necessary step in distal AVF creation. After the mobilization of vessels for an appropriate distance (around 3 cm), microvascular clamps were applied to perform end-to-side or side-to-side anastomoses. Arteriotomy of 12 mm was done in cases of side-to-side anastomosis and 6 mm in the case of end-to-side anastomosis. An end-to-end anastomosis was performed when a good caliber branch of the radial artery was found or if the radial artery was severely atherosclerotic. In all other cases, an end-to-side anastomosis was done. Staining of the arteriotomy site was done using methylene blue to facilitate easy differentiation between the adventitial and endothelial layers. The cephalic vein was dissected proximally for a distance of 3 cm to reduce acute turns or twists that may result in turbulence after the completion of the anastomosis.

B) Proximal Brachiocephalic AVF

End-to-side anastomosis is performed between the antecubital or cephalic vein and the brachial artery. The antecubital vein was preferred for anastomosis if available. The first valve towards the distal limb was broken using a vessel dilator before anastomosis. This allows the veins in the distal forearm to develop simultaneously along with proximal veins, giving an increased length of the arterialized vein for cannulation.

Skin closure was done with 4-0 Ethilon in a single layer. The non-compressive dressing was given. Bruit was heard and thrill was felt on the operation table at the end of the procedure. Instructions about how to feel the thrill were given and patients were asked to report signs of any coldness, numbness, ulcers, or discoloration of the fingertips.

C) Brachiobasilic AVF (BBAVF)

We performed BBAVF as a one-stage transposition technique. The details are provided below:

1. A straight-line incision was made over the medial aspect of the arm with a curvilinear extension just distal to the ante-cubital fossa.

2. Basilic vein was dissected along the entire length up to the axilla and was also separated from the medial cutaneous nerve of the forearm.

3. Once the dissection was complete, the basilic vein was cut distally, thereby ensuring the availability of adequate length to perform AV anastomosis without tension once the vein was transposed subcutaneously through the tunnel.

4. Transposition of the basilic vein through the subcutaneous tunnel over the arm was performed.

5. End-to-side brachiobasilic anastomosis was performed.

6. Wound closure was done in layers.

The postoperative protocol followed at our institution is laid out in Table 2

**Table 2:** Postoperative protocol

Postoperative protocol
Tab amoxicillin-clavulanic acid 625 mg twice a day for 5 days
Tab pantoprazole 40 mg once a day for 5 days
Tab paracetamol 500 mg SOS if pain persists for 5 days
Tab aspirin 75 mg once a day for 15 days
Left/right upper limb elevation
Handball exercises/elbow exercises after 24 hours (to be continued)
Avoid dialysis for the next 24 hours
Heparin-free dialysis for one week
Watch for bleeding; if bleeding occurs, report stat
Do not take blood pressure measurements from the fistula arm
Do not have any blood tests taken from the fistula arm
No needles, infusions, or drips go into the fistula arm
Do not wear any tight or restrictive clothing on the fistula arm
Avoid sleeping on the fistula arm
Do not use sharp objects near the fistula arm, e.g., razors
Avoid carrying heavy loads or shopping bags directly over the fistula
Do not remove the scabs from the needle sites as this may cause bleeding or an infection
Fistula can be used for dialysis after 6 weeks
Follow up after two days for wound check and after 10 days for suture removal

Data analysis

The data were analyzed using SPSS Statistics version 20 (IBM, Armonk, NY). Age, gender, comorbidities like diabetes, type of anastomosis, and arterial and vein diameters were evaluated to ascertain their impact on the outcome of fistula creation (successful and unsuccessful).

The student's unpaired t-test was used to compare the outcomes of age and arterial and vein diameter. Data were represented as mean ± standard deviation. A p-value of <0.05 was considered statistically significant. Similarly, the Chi-square test was used to compare outcomes of gender and diabetes. The degree of freedom for both was one and a p-value of <0.05 was considered statistically significant. Regression analysis was used to find predictors or risk factors (age, gender, presence or absence of diabetes, type of anastomosis, and arterial and vein diameters) for a successful outcome.

## Results

In our cohort of 216 patients, the average age at presentation was 43.9 years. Among them, 34.6% were females and 65.74% were males. The difference in mean age between the successful and unsuccessful outcome groups was insignificant (p=0.825). The successful outcome in males was higher than that in females (p=0.0054). The proportion of patients with diabetes in our study was 16.67%, and its incidence rate among successful and unsuccessful groups was similar and statistically insignificant (Tables 3, 4, 5).

**Table 3:** Mean age of both outcome groups SD: standard deviation

Success	Age in years (mean ± SD)	Test	P-value
Yes (n=197)	43.8 ± 14.9	Unpaired t-test	0.8253
No (n=19)	44.6 ± 16.2		

**Table 4:** Arteriovenous fistula outcomes in males and females Chi-square test p=0.005

Sex	Arteriovenous fistula outcome		
	Successful	Unsuccessful	Total
Females (n)	62	12	74
Proportion	83.78%	16.22%	100.00%
Males (n)	135	7	142
Proportion	95.07%	4.93%	100.00%
Total (n)	197	19	216
Proportion	91.20%	8.80%	100.00%

**Table 5:** Arteriovenous fistula outcome in diabetics Chi-square test p=0.91

Diabetes	Successful outcome		
	Yes	No	Total
Yes (n)	33	3	36
Proportion	91.67%	8.33%	100.00%
No (n)	164	16	180
Proportion	91.11%	8.89%	100.00%
Total (n)	197	19	216
Proportion	91.20%	8.80%	100.00%

The mean arterial diameter in the successful group was marginally greater than that in the other group, but this was statistically insignificant (p=0.225). The mean vein diameter of the successful group was significantly larger than that in the unsuccessful group (p=0.0004) (Table 6).

**Table 6:** Mean artery and vein diameter of both outcome groups SD: standard deviation

Successful outcome	Vein diameter (mean ± SD)	Test	P-value
Yes (n=197)	2.4 ± 0.7	Unpaired t-test	0.0004
No (n=19)	1.9 ± 0.5		
	Artery diameter (mean ± SD)		
Yes (n=197)	2.5 ± 0.5	Unpaired t-test	0.2225
No (n=19)	2.3 ± 0.4		

**Figure 1:**
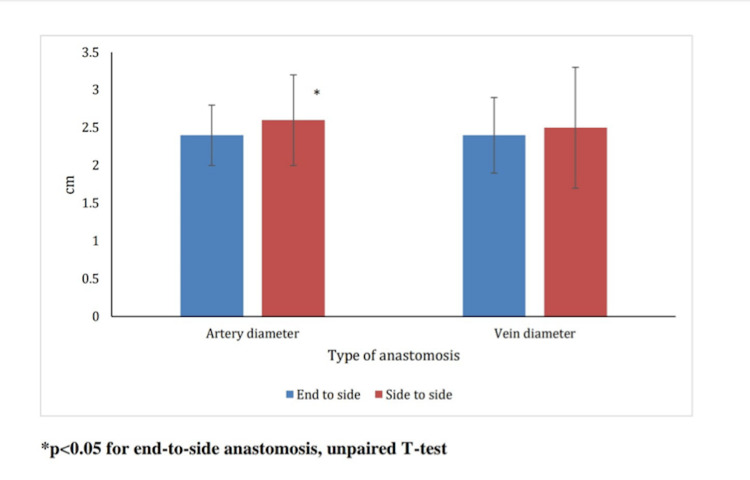
Comparison of the mean artery and vein diameter in end-to-side vs. end-to-end anastomosis

End-to-side anastomosis had a successful outcome in 92.7% of cases vs. 88.3% in side-to-side anastomosis. The mean arterial diameter for an end-to-side anastomosis was 2.4 mm and that for side-to-side anastomosis was 2.6 mm. This was statistically significant (p=0.01). The mean venous diameter for an end-to-side anastomosis was 2.4 mm and that for side-to-side anastomosis was 2.5 mm, but this was not statistically significant (Figure 1).

In both males and females, outcomes in the clerical group were comparable to the laborer group. The distal arterial diameter was higher in both males and females in the laborer group, which was statistically significant (p=0.03 and 0.05 respectively). Distal vein diameter was also found to be higher in males and females among laborers but this was not statistically significant (p=0.35 and 0.34 respectively) (Tables 7, 8).

**Table 7:** Mean artery and vein diameter of distal arteriovenous fistula in males SD: standard deviation

Group	Vein diameter (mean ± SD)	Test	P-value
Clerical (n=197)	2.3 ± 0.6	Unpaired t-test	0.35
Laborer (n=19)	2.5 ± 0.6		
	Artery diameter (mean ± SD)		
Clerical (n=197)	2.2 ± 0.3	Unpaired t-test	0.03
Laborer (n=19)	2.4 ± 0.3		

**Table 8:** Mean artery and vein diameter of distal arteriovenous fistula in females SD: standard deviation

Group	Vein diameter (mean ± SD)	Test	P-value
Clerical (n=21)	1.9 ± 0.5	Unpaired t-test	0.34
Laborer (n=78)	2.1 ± 0.5		
	Artery diameter (mean ± SD)		
Clerical (n=21)	2.2 ± 0.3	Unpaired t-test	0.05
Laborer (n=78)	2.4 ± 0.3		

The overall complication rate in our study was 22.22%. The most common complications were distal limb edema (10.3%) and thrombosis (8.7%). Other complications included aneurysm formation, bleeding due to clip slippage, hematoma formation, and perioperative cellulitic changes (Table 9).

**Table 9:** Summary of the complications in our study

Complications	Frequency	Percentage
Aneurysm formation	1	0.46%
Bleeding from clip slippage	1	0.46%
Edema	22	10.19%
Hematoma formation	3	1.39%
Perioperative cellulitic changes - resolved	1	0.46%
Postoperative bleeding due to slippage of clip	1	0.46%
Thrombosis	19	8.80%
No complication	168	77.78%
Total	216	100.00%

In 203 (93.9%) out of 216 cases, bruit was present at the end of surgery. Results showed 90% and 83% patency of fistulas at the end of six months and one year respectively. The primary failure rate was 8.80%.

## Discussion

Currently, HD treatment provides a life-saving option for patients with CKD and enables them to survive for many years with a good quality of life [3]. Improved life expectancy associated with HD has created a demand for a reliable VA. Central venous catheters are the primary method of choice for temporary access in cases where there is an urgent need for HD and no other VA is available or has failed [2]. However, these devices are associated with several complicating factors such as infection, thrombosis, venous stenosis, and damage to proximal vessels. This has intensified the demand for a reliable and safe VA.

Earlier, VA was achieved through permanent implantation of a cannula into an artery and vein of a forearm. Between dialysis, patency of these blood vessels was maintained through external Teflon-Silastic/polytetrafluoroethylene (PTFE) tubes creating an external AVF. A higher overall complication rate and morbidity linked to these external fistulas led to the development of an internal AVF [4]. In 1966, Brescia and Cimino published their ingenious method of a forearm AVF, but they selected relatively young patients for their study [4].

The ideal VA should provide adequate flow rates to sustain dialysis, be easy to access/cannulate, be cost-effective, and have excellent long-term patency and minimal complications [2]. Hence, native AVF is the VA of choice [2,5]. The NKF-DOQI Clinical Practice Guidelines encourage the creation of primary AV fistulae for the majority of new patients elected to receive long-term HD [2].

Patients who have received dialysis across a functional AVF have low complication rates and a longer duration of event-free patency than patients with catheter access and AV grafts (AVGs) [6]. Thus, a native AVF is a more reliable and safer option over prosthetic grafts and central venous catheters.

The main drawback of AVF is the non-maturation (failure to use the new fistula for successful HD sessions) as the newly formed conduit needs to mature into a low-resistance circuit with increased flow rates. A non-matured AVF is one that becomes non-usable for dialysis within three months of its creation [6]. The NKF-DOQI guidelines recommended the "rule of sixes" to define fistula maturation. This includes a flow rate of 600 ml/min, AVF being located 6 mm below the skin surface, and a minimal fistula diameter of 6 mm. A mature fistula should allow a flow rate of at least 300-400 ml/min [1,2,7].

We performed AVF in all CRF patients as the first choice of VA. After a thorough history and clinical examination, Doppler USG was performed in all cases. If vessel condition was not good or the caliber was small, we preferred performing a more proximal fistula to improve the chances of success and reduce the morbidity related to undergoing repeated procedures and delay in initiating HD.

Among the total 216 cases, the difference in mean age between groups was insignificant (p=0.825), showing that age did not contribute to successful outcomes. We found that the outcome among males was more successful (p=0.0054). This probably correlated with their work profile. Most of the females were housewives or did desk jobs while males were involved in laborious work or occupations that required heavy use of arms/forearms. Hence, this may be a surrogate indicator for the impact of preoperative forearm/arm exercises on the outcomes of AVF creation.

Diabetes represents an ever-growing cause of end-stage renal disease (ESRD). In the past, even though diabetes was considered a risk factor for fistula non-maturation, several studies have reported successful outcomes in diabetic patients. The proportion of diabetic patients in our study was 16.67%, which was significantly lower compared to other studies [8,9]. Sedlacek et al. reported that diabetes was not an independent risk factor for AVF non-maturation, and the presence of diabetes did not affect the success of AVF creation [10]. Allon et al. reported that both diabetes and age did not influence AVF maturation outcomes, although both were significantly linked to increased intimal hyperplasia. But the sample size of this study was significantly small with 50% being female and 50% affected by diabetes [9]. Similarly, Farber et al. found that diabetes was not associated with early thrombosis [11]. On the contrary, Salmela et al. found that diabetes mellitus, female sex, and thrombophilia were associated with decreased primary fistula patency rates [8]. In our study, we found that outcomes in patients with or without diabetes were similar (p=0.914), suggesting that diabetes was not an independent risk factor for the non-maturation of AVF. But in patients with multiple comorbidities and in the presence of other negative predictors, it might have an impact on the outcome.

The mean arterial diameter in the successful group was marginally greater than that in the other group (p=0.225). However, the mean diameter of the vein was significantly greater than that in the other group (p=0.0004). The American Institute of Ultrasound in Medicine Practice Guidelines 2016 state that arterial diameter <2 mm and venous diameter <2.5 mm were associated with a high failure rate [12]. Our study also proves that venous diameters <2 mm are associated with significantly higher non-maturation rates (p<0.001). Since the mean arterial diameter was around 2.4 mm in our study, the impact of arterial diameter being <2 mm could not be commented upon. The type of anastomosis had no significant association with the AVF outcome.

This is the first study to assess the role of patient occupation on the outcomes of AVF creation and maturation. We observed that males and females engaged in manual labor had a greater artery and vein caliber as compared to the clerical group. This may indirectly demonstrate the benefit of preoperative forearm exercises on AVF maturation. Forearm exercises sustainably improve cephalic vein diameter and volume within four to six weeks and guidelines recommend regular forearm and arm exercises after AVF creation [13-16]. However, the outcomes in the clerical and laborer group were similar in our study, probably due to skewed distribution among both genders. Further randomized trials are required to evaluate the influence of occupation on the final outcomes.

It is common practice to aim for an RCAVF at the wrist [17]. However, rates of RCAVF primary non-maturation are as high as 30-60% [18,19]. Due to greater vessel caliber, people performing heavy labor can be considered for snuffbox AVF/distal AVF despite higher rates of non-maturation in distal AVF in comparison to people with clerical occupation who might benefit from a proximal AVF. In our study, all patients in the laborer group had a successful creation of distal AVF with no requirement for proximal fistula in the one-year follow-up period. Also, the creation of a distal AVF decreases the chance of HD-induced distal ischemia (HAIDI) as well as cardiac complications and preserves the possibility of proximal AVF creation [20].

The distal forearm was the most common site for the distal radiocephalic fistula. It accounted for 139 (64.35%) of our operative procedures. In our analysis of 139 (64.35%) distal fistulas, 126 (90.64%) were successful. High radiocephalic (mid-forearm), brachiocephalic, and brachiobasilic AVFs were reserved for patients with previously failed Brescia-Cimino AVFs or in cases where appropriate vessels were not available for anastomosis at the wrist level.

The reported incidence of primary failure in medical literature varies from 20 to 60% [12,21-23], which is comparable with our results of a 9.36% primary failure rate. Adherence to a stepwise perioperative approach, the use of magnification, and strict follow-up contributed to the overall successful outcome in our study.

We performed six brachiobasilic and 30 brachiocephalic fistulas in our series. Out of these 36 procedures, only three brachiocephalic fistulas were unsuccessful. We performed brachiobasilic fistula as a single-stage transposition procedure. This requires bringing basilic vein from the deeper compartment to a more superficial location in order to facilitate easy cannulation. This also prevents injury to the medial antebrachial cutaneous (MABC) nerve [24]. All brachiobasilic fistulas were successful with postoperative edema being the most common complication. Our dialysis technicians were also very satisfied with the ease of cannulation in the single-stage transposition AVF. Hossny, in a series of 70 patients with brachiobasilic fistulas, observed that all dialysis nurses were satisfied with the transposed veins, but only 53.3% were satisfied with the elevated veins; the difference was statistically very significant (p<0.001) [25]. He summarized that while different techniques for creating brachiobasilic AVF produced no difference in the patency rate, this was not the case with complications. Complications in the elevated vein group were greater than those in the transposed vein group.

We established a direct correlation of bruit heard and thrill felt at the end of the surgery with the outcome of the fistula. In cases where both were absent, the failure rate was as high as 57%. In case there was no thrill or bruit at the end of two weeks, we preferred to perform a new proximal fistula. Kazemzadeh et al., in their case series of 245 patients, had primary patency at six months and one and two years as follows: 79.5%, 70%, and 65% [26]. This was comparable to our results that showed 90% and 83% patency of fistulas at the end of six months and one year respectively. Sultan et al. observed that at four years, primary functional patency was better with proximal fistula as compared with distal fistulas [27].

In our study, we found that only the vein diameter was predictive of successful outcomes (Table 10).

**Table 10:** Predictive factors of successful outcomes after arteriovenous fistula creation

Variable	Coefficient	Standard error	P-value	Odds ratio	95% confidence interval
Age	-0.0147	0.0205	0.4733	0.9854	(0.9465-1.0258)
Gender	-0.9751	0.5352	0.0685	0.3772	(0.1321-1.0767)
Diabetes mellitus	0.3347	0.7574	0.6586	1.3975	(0.3167-6.1661)
Type of anastomosis	-0.3768	0.4924	0.4442	0.6861	(0.2613-1.8010)
Artery	-0.3779	0.5919	0.5232	0.6853	(0.2148-2.1863)
Vein	1.8552	0.6415	0.0038	6.3932	(1.8183-22.4780)
Constant	1.9065	2.0145	0.3440		

Factors like age and life expectancy, gender, comorbidities, condition of vessels, and type of anastomosis may not be individually predictive of the outcome but need to be considered before choosing the appropriate site of access creation according to the life plan of the patient.

Our study is not without limitations. It was a retrospective analysis of the factors that might be helpful in predicting successful outcomes of AVF creation. Certain parameters like depth of vessels from the skin surface and clinical laboratory parameters were not included, and hence their role or bias could not be determined. A well-designed randomized control trial that takes into account the above-mentioned factors might be more predictive and informative in guiding the choice of AVF creation. Also, conducting a study with a larger sample size will be more representative of the general population.

## Conclusions

Based on our findings, age and life expectancy of the individual, male gender, occupation as a laborer, associated comorbidities, and vessel condition are important clinical predictive factors that can guide the choice of the location for AVF creation, which in turn will help in creating a successful AVF. This will reduce the morbidity associated with an additional procedure and enable the initiation of HD as early as possible. Patient occupation can be considered as a surrogate for preoperative forearm exercises with the increased caliber of vessels found in people performing heavy/manual labor favoring more distal AVF creation.

## References

[1] Bashar K, Conlon PJ, Kheirelseid EA, Aherne T, Walsh SR, Leahy A (2016). Arteriovenous fistula in dialysis patients: Factors implicated in early and late AVF maturation failure. Surgeon.

[2] Foster BJ, Mitsnefes MM, Dahhou M, Zhang X, Laskin BL (2018). Changes in excess mortality from end stage renal disease in the United States from 1995 to 2013. Clin J Am Soc Nephrol.

[3] Lok CE, Huber TS, Lee T (2020). KDOQI clinical practice guideline for vascular access: 2019 update. Am J Kidney Dis.

[4] Brescia MJ, Cimino JE, Appel K, Hurwich BJ (1966). Chronic hemodialysis using venipuncture and a surgically created arteriovenous fistula. N Engl J Med.

[5] Vassalotti JA, Jennings WC, Beathard GA, Neumann M, Caponi S, Fox CH, Spergel LM (2012). Fistula first breakthrough initiative: targeting catheter last in fistula first. Semin Dial.

[6] Dhingra RK, Young EW, Hulbert-Shearon TE, Leavey SF, Port FK (2001). Type of vascular access and mortality in U.S. hemodialysis patients. Kidney Int.

[7] Gilmore J (2006). KDOQI clinical practice guidelines and clinical practice recommendations--2006 updates. Nephrol Nurs J.

[8] Salmela B, Hartman J, Peltonen S, Albäck A, Lassila R (2013). Thrombophilia and arteriovenous fistula survival in ESRD. Clin J Am Soc Nephrol.

[9] Allon M, Litovsky S, Young CJ (2011). Medial fibrosis, vascular calcification, intimal hyperplasia, and arteriovenous fistula maturation. Am J Kidney Dis.

[10] Sedlacek M, Teodorescu V, Falk A, Vassalotti JA, Uribarri J (2001). Hemodialysis access placement with preoperative noninvasive vascular mapping: comparison between patients with and without diabetes. Am J Kidney Dis.

[11] Farber A, Imrey PB, Huber TS (2016). Multiple preoperative and intraoperative factors predict early fistula thrombosis in the Hemodialysis Fistula Maturation Study. J Vasc Surg.

[12] Allon M (2007). Current management of vascular access. Clin J Am Soc Nephrol.

[13] Wilschut ED, Rotmans JI, Bos EJ, van Zoest D, Eefting D, Hamming JF, van der Bogt KE (2018). Supervised preoperative forearm exercise to increase blood vessel diameter in patients requiring an arteriovenous access for hemodialysis: rationale and design of the PINCH trial. J Vasc Access.

[14] Oder TF, Teodorescu V, Uribarri J (2003). Effect of exercise on the diameter of arteriovenous fistulae in hemodialysis patients. ASAIO J.

[15] Leaf DA, MacRae HS, Grant E, Kraut J (2003). Isometric exercise increases the size of forearm veins in patients with chronic renal failure. Am J Med Sci.

[16] (2021). NKF KDOQI Clinical Practice Guidelines and Clinical Practice Recommendations. 2006 updates. http://www.kidney.org/professionals/KDOQI/guidelines.

[17] Tordoir J, Canaud B, Haage P (2007). EBPG on vascular access. Nephrol Dial Transplant.

[18] Dember LM, Beck GJ, Allon M (2008). Effect of clopidogrel on early failure of arteriovenous fistulas for hemodialysis: a randomized controlled trial. JAMA.

[19] Rothuizen TC, Wong C, Quax PH, van Zonneveld AJ, Rabelink TJ, Rotmans JI (2013). Arteriovenous access failure: more than just intimal hyperplasia?. Nephrol Dial Transplant.

[20] Dillard MG, Alexander PC (1979). High output cardiac failure secondary to a Brescia-Cimino fistula. J Natl Med Assoc.

[21] Allon M, Robbin ML (2002). Increasing arteriovenous fistulas in hemodialysis patients: problems and solutions. Kidney Int.

[22] Cheung AK, Imrey PB, Alpers CE (2017). Intimal hyperplasia, stenosis, and arteriovenous fistula maturation failure in the hemodialysis fistula maturation study. J Am Soc Nephrol.

[23] Lok CE (2007). Fistula first initiative: advantages and pitfalls. Clin J Am Soc Nephrol.

[24] Wang S, Wang MS, Jennings WC (2017). Basilic elevation transposition may improve the clinical outcomes for superficialization of basilic arteriovenous fistula veins. J Vasc Surg.

[25] Hossny A (2003). Brachiobasilic arteriovenous fistula: different surgical techniques and their effects on fistula patency and dialysis-related complications. J Vasc Surg.

[26] Kazemzadeh GH, Modaghegh MHS, Ravari H, Daliri M, Hoseini L, Nateghi M (2012). Primary patency rate of native AV fistula: long term follow up. Int J Clin Exp Med.

[27] Sultan S, Hynes N, Hamada N, Tawfick W (2012). Patients on hemodialysis are better served by a proximal arteriovenous fistula for long-term venous access. Vasc Endovascular Surg.

